# Surgical Treatment of Type 31-A1 Two-part Intertrochanteric Femur Fractures: Is Proximal Femoral Nail Superior to Dynamic Hip Screw Fixation?

**DOI:** 10.7759/cureus.4110

**Published:** 2019-02-20

**Authors:** Hariharan Mohan, Prakash Kumar

**Affiliations:** 1 Orthopaedics, Rajawadi Municipal Hospital, Mumbai, IND

**Keywords:** intertrochanteric fracture, proximal femur nail, dynamic hip screw, functional outcome

## Abstract

Introduction

The implant of choice for two-part intertrochanteric femur fracture is still under debate. This study was done to compare the operative parameters and functional outcome of two-part intertrochanteric fractures treated by dynamic hip screw (DHS) and proximal femur nail (PFN).

Methods

Fifty-four operated cases of two-part intertrochanteric (AO 31A1) were analysed and divided into two groups based on implant used (PFN 30, DHS 24). Operative details, which include blood loss and duration of surgery, were obtained from hospital records. All patients were followed up for six months and assessed for radiographic and functional outcome. The functional outcome was calculated with modified Harris hip score and Parker mobility score.

Results

There was no significant difference in the operative parameters (p > 0.05) between DHS and PFN. The average blood loss for DHS and PFN was 202.5 ml and 198 ml respectively while operative duration was 136 min and 126 min, respectively. All patients had good functional outcome at the end of six months with average Harris hip score of 69.7 and Parker score of 8. No difference was found between the two surgeries in terms of functional outcome as well (p > 0.05).

Conclusion

There is no conclusive evidence to show that PFN is superior to DHS in the treatment of two-part intertrochanteric (31A1) fracture. Both DHS and PFN are equally effective in treatment of such fractures.

## Introduction

Intertrochanteric fractures account for nearly 50% of all fractures of the proximal femur [1]. They have the highest postoperative fatality rate of surgically treated fractures. Intertrochanteric fractures are classified as stable and unstable based on the intactness of posteromedial cortex and amount of comminution. Both these fracture patterns are globally viewed as an injury best treated with surgical repair. The dynamic hip screw remains the primary mode of fixation of these fractures. Recently its usage has declined due to its complications. These include uncontrolled collapse and migration of the lag screw within the femoral head leading to varus collapse and screw cutout. The incidence of this is increased in malreduced fractures or those with iatrogenic fracture of the lateral wall. To circumvent these issues, the proximal femoral nails were introduced. In recent years, the usage of PFN has greatly increased as it is considered to be associated with decreased operative complications and better functional outcome. Various studies have been done comparing the outcome of DHS and PFN in intertrochanteric fractures. Studies in the past have showed no difference in outcome between the two, but recent studies suggest that PFN is a better implant for complex unstable intertrochanteric fractures with better outcomes and less complications [2,3]. The choice of implant for simple two-part fractures (31A1) is still under debate. There are very few studies in literature comparing PFN and DHS in treatment of stable two-part intertrochanteric fractures. In this study, we have compared the functional outcome of stable two-part (31A1) intertrochanteric femur fractures treated with DHS and PFN. We have also compared the intra-operative and radiological features between the two surgeries.

## Materials and methods

This retrospective study was conducted in our institute between May 2017 and June 2018. Data of patients with intertrochanteric femur fracture operated during the study period was analysed. Fifty-four patients with simple two-part intertrochanteric fracture (type 31A1) were included in the study. Twenty-four patients had undergone fixation with DHS while the remaining 30 were fixed with PFN. Complex, comminuted intertrochanteric fracture (type 31A2, 31A3), polytrauma patients and patients with neurological deficit were excluded from the study. Pre-operative and intra-operative data were collected from hospital records. These include patient demographics, duration of surgery, blood loss and post-operative X-ray. Operative time was calculated from incision time to closure. Patients were assessed during their follow-up visit to the hospital six months post surgery. Antero-posterior and lateral radiographs of the operated hip were done and patients were clinically assessed for limb length discrepancy and functional outcome. The following parameters were assessed in follow-up X-ray: (1) Fracture union: appearance of bridging callus and disappearance of fracture line, (2) Screw protrusion or cut-out, (3) Peri-implant fracture. Functional outcome was assessed at the end of six months and calculated using the modified Harris hip score (score 0-91) and Parker mobility score (scores 0-9) [4,5].

Comparisons were conducted on all study variables to determine whether there were any differences between the two surgical procedures. Study variables were analysed and described with means and standard deviations. Student t-test for continuous variables with normal distributions, and Mann–Whitney test for variables with skewed distributions were used to compare the variable between the two surgeries. A p-value of less than 0.05 was considered to be statistically significant.

## Results

After applying exclusion criteria, 54 patients were included in our study. All patients had AO type 31A1 fracture that was treated surgically. Dynamic hip screw was used in 24 patients and the remaining had proximal femur nail fixation. All patients were operated under regional anaesthesia (spinal ± epidural) with fluoroscopic guidance in supine position in a fracture table. Closed reduction and internal fixation were performed in all cases. DHS implant used included four hole long or short barrel plate (130°, 135°) and Richard screw of suitable length. PFN used included 180 and 250 mm nails (130°, 135°) with two cephalomedullary screws (8 mm, 6.5 mm) and one or two distal locking bolts. Partial weight bearing mobilisation was started in the immediate post-operative period for all patients and full weight bearing as per tolerance was started after six weeks. All patients were assessed during their follow-up visit after six months.

Among the 54 patients, 34 were males and 20 were females. The youngest patient was 25 years of age and the oldest was 92 years with the mean age being 53 years (±17). DHS was used predominantly in younger and middle age groups while PFN was used predominantly in older age groups. The average age of the patient with DHS was 45 (±14) years while that for PFN was 60 (±16) years (Table 1).

**Table 1:** Patient demographics. DHS: Dynamic hip screw; PFN: Proximal femur nail.

Surgery	Total	Male	Female	Age (years)			
				20-40	41-60	>61	Mean
DHS	24	16	8	9	9	6	45 (±14)
PFN	30	18	12	4	8	18	60 (±16)

The mean blood loss during surgery for the entire study group was 200 ml with the average blood loss for DHS and PFN group being 202.5 and 198 ml, respectively. Statistical analysis (t-test) showed no significant difference between the intra-operative blood loss between the two modes of surgery (p- 0.5716). Similar results were seen in terms of duration of surgery where the mean operative duration was 130 min, 136 min and 126 min for the entire study group, DHS group and PFN group, respectively. Statistical analysis showed no significant difference in the duration of surgery between the two groups (p- 0.257).

Two patients required second surgery during the follow-up period. One patient with DHS developed infection, which settled with debridement, wound wash and intravenous antibiotics. The other patient was a patient with PFN who required removal of backed out screw due to reverse-Z effect. The same patient had lateral thigh pain which was relieved with screw removal. All patients were able to walk with or without support at six months. Twenty patients were able to squat and sit cross-legged. Functional outcome of the patient was calculated using the Parker mobility score and modified Harris hip score. The mean Parker score for the entire study population was 7.77. DHS and PFN group had mean scores of 8 and 7.6, respectively. There was no significant difference in the outcome at six months between the two procedures as the p-value was 0.9203. Similarly the mean Harris hip score of DHS group was 70.6 and that of PFN group was 69. Statistical test (Mann–Whitney) showed no difference (p- 0.568) in the functional outcome between the two modes of surgeries (Table 2).

**Table 2:** Variables compared between dynamic hip screw (DHS) and proximal femur nail (PFN).

Factor	DHS	PFN	p-value
Blood loss (ml)	202.5 (±22.12)	198 (±33.26)	0.5716
Operative duration (min)	136 (±24)	126 (±37)	0.2572
Modified Harris hip score	70.6 (±4.08)	69 (±5.52).	0.9203
Parker mobility score	8 (±0.72)	7.6 (±1.7)	0.5686
Complications			
1. Infection	1	0	
2. Screw Back-out	0	1	

**Figure 1:**
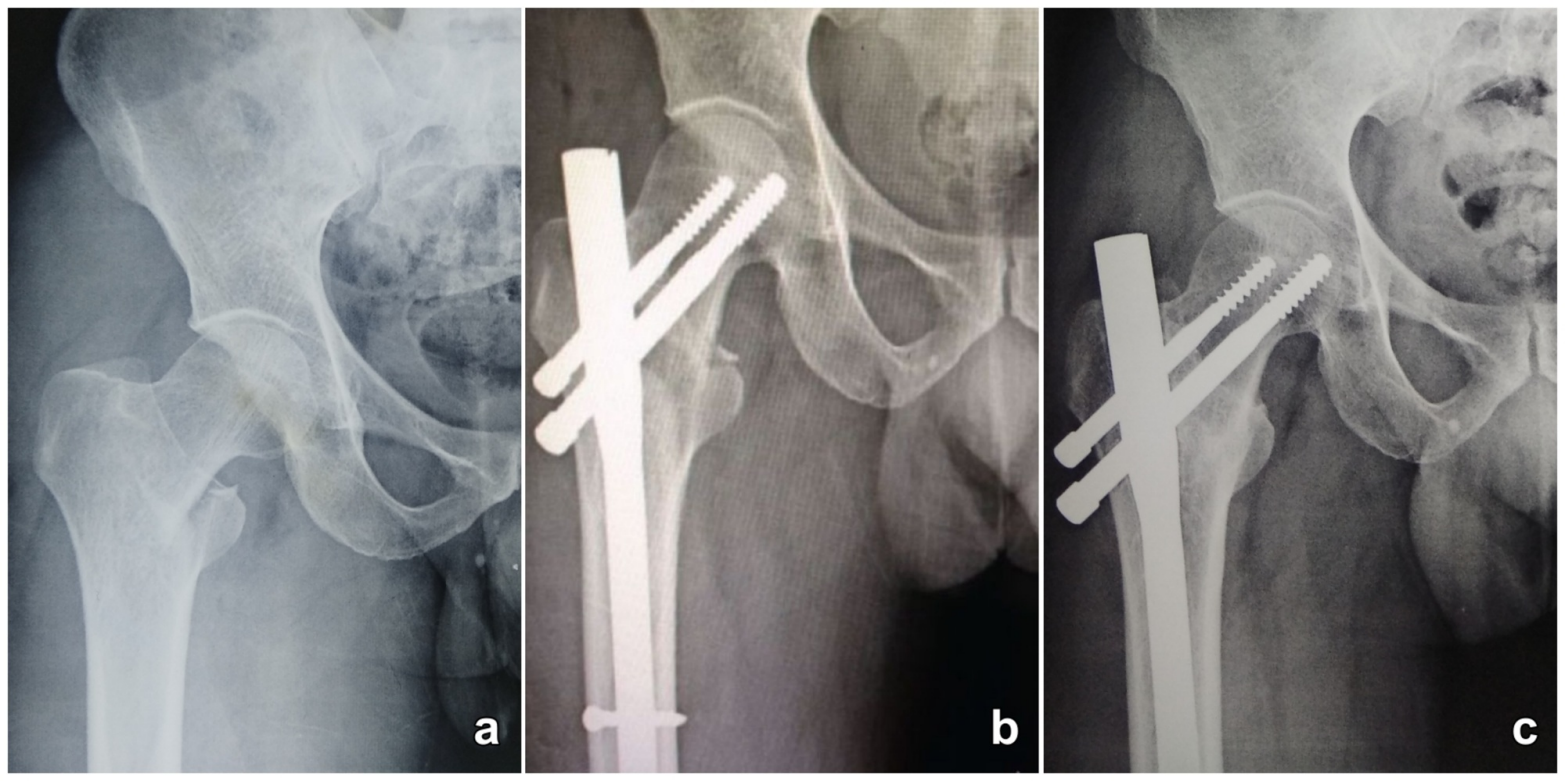
Radiograph of 45-year-old male with 31A1 intertrochanteric fracture fixed with proximal femur nail. (a) Pre-operative radiograph showing two-part fracture. (b) Radiograph taken on day 1 post-surgery. (c) Radiograph taken six months post-surgery showing complete union of fracture.

**Figure 2:**
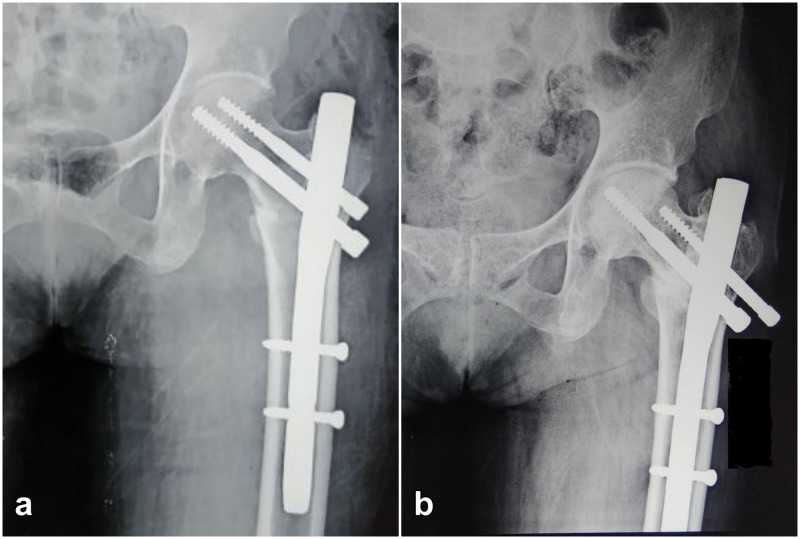
Radiograph of 65-year-old female patient with screw back-out. (a) Radiograph taken on day 1 post-surgery. (b) Radiograph taken six months post-surgery showing reverse-Z effect and fracture union.

Analysis of radiographs at six months showed union of fracture in all 54 cases (Figure 1). One patient with PFN had screw back-out due to reverse-Z effect (Figure 2). The neck-shaft angle of all patients was between 125 to 140 degrees. Eighteen out of 30 patients with PFN had their implant protruding out of the greater trochanter and not in line with GT tip. But no discomfort was perceived by the patients due to this within the six-month period. None of the patients with DHS has any implant prominence.

## Discussion

In this study, we have compared the outcome of DHS and PFN done in AO type 31A1 fractures. AO 31A1 includes simple two-part fractures of the pertrochanteric area with A1.1 fractures along the intertrochanteric line, A1.2 fractures through the greater trochanter and A1.3 fractures below the lesser trochanter. All these are stable fractures with an intact posteromedial cortex.

The mean age of the study population is 53 (±17) years, which is lesser than the usual age of incidence of intertrochanteric fractures, which is 66-76 years [6]. The average age of the study population of previous studies by Pajarinen et al. and Parker et al. was 80 years [7,8]. This can be attributed to the fact that only simple two-part fractures were included in our study. Older people with osteopenic bone are usually associated with unstable comminuted fractures while stable fractures are more common in the younger age group. This also correlates with the increased number of male patients in our study (1.7:1). Usual male:female ratio of IT fractures is 1:3,4 [6]. The increased incidence in females is attributed to postmenopausal osteoporosis, which is usually associated with unstable fractures. Hence our study with two-part fractures had predominantly male patients.

Our study showed no difference in the intra-operative features between DHS and PFN. The factors considered were intra-operative blood loss and operative duration. The average blood loss for DHS and PFN groups was 202.5 ml and 198 ml, respectively. This result was similar to several studies in literature. Pajarinen et al. studied outcome of 108 operated cases of PFN and DHS (all AO type fractures) and found average blood loss was 320 ml and 357 ml respectively for the two groups and no statistical difference between the two groups [7]. Recent meta-analysis by Zhang et al. in 2018 showed that there was no significant difference in the blood loss and requirement of blood transfusion between the two surgeries [3].

There was no significant difference in operative time between the two surgeries (mean PFN 126 min, mean DHS 136 min). This is similar to results available in literature. Giraud et al. studied 60 patients and found that the average operative time for PFN and DHS was 35 and 42 min, respectively, with no significant statistical difference [9]. Meta-analysis by Huang et al. in 2013 showed that there was no significant difference in the operative time between DHS and PFN [10]. They concluded that operative time depends upon the skill of the surgeon and his experience with using the specific implant. All these studies included both stable and unstable intertrochanteric fractures for comparison of results.

The functional outcome was assessed at the end of six months with modified Harris hip score and Parker mobility score. Good outcome was seen in all 54 patients. There was no statistical difference in functional outcome between the two surgeries. This study is one of the first studies to compare PFN and DHS in two-part intertrochanteric fractures. Zeng et al. in 2017 compared the outcome of PFN-antirotation and DHS in AO 31A1 fractures and showed that PFN-A group had better outcome and lesser radiographic complications compared to DHS group [2]. Older studies comparing Gamma nails or Targon nails with DHS showed no difference in functional outcome [11,12]. However, recent studies comparing PFN antirotation nails with DHS have shown that PFN is better for unstable intertrochanteric fractures while there is no significant difference in case of stable fractures [3,13,14] (Table 3).

**Table 3:** Comparison of results of our study with similar studies in literature. * Meta-analysis PFN: Proximal femur nail; DHS: Dynamic hip screw.

Study	Year	Fracture type	Implants compared	Mean age (years)	Mean follow-up (months)	Factors compared	Result
Pajarinen et al. [7]	2004	31A1, 31A2, 31A3	PFN, DHS	80	4	Blood loss	No difference
						Operative time	PFN > DHS
						Radiological outcome	No difference
Parker et al. [8]	2012	31A1, 31A2, 31A3	Targon PFN, DHS	82	12	Blood loss	No difference
						Operative time	PFN > DHS
						Complication rate	No difference
						Return to mobility	PFN better than DHS
Huang et al.* [10]	2013	31A1, 31A2, 31A2	PFN, DHS	-	3-12	Blood loss	No difference
						Operative time	No difference
						Complication rate	No difference
Zhang et al.* [14]	2014	31A1, 31A2, 31A3	PFN, DHS	-	3-12	Blood loss	DHS > PFN
						Operative time	DHS > PFN
						Complication rate	No difference
Zeng et al. [2]	2017	31A1	PFN-A, DHS	75	12	Radiological outcome	PFN better than DHS
						Functional outcome	
Zhang et al.* [3]	2018	31A2, 31A3	PFN, DHS	-	12	Mortality	No difference
						Functional outcome	PFN better than DHS
						Complication rate	DHS > PFN
Our study	2018	31A1	PFN, DHS	53	6	Blood loss	No difference
						Operative time	
						Functional outcome	
						Radiological outcome	

In our study, all fractures showed union at the end of six months. These excellent results can be attributed to anatomical reduction that was achieved intra-operatively in all cases. The incidence of complications for both DHS and PFN is more in case of malreduced fractures. Protrusion of PFN implant over greater trochanter tip, which was seen in 18 patients, was not associated with any discomfort. Longer follow-up might be required to study the actual effect of this implant prominence. Dodenhoff et al. in 1997 showed that prominence of the nail proximally was not associated with pain, but protuberance of laterally-based proximal locking screws caused problems like proximal thigh pain [15].

## Conclusions

We conclude that both PFN and DHS are equally effective in the treatment of stable two-part intertrochanteric femur fractures. They show no difference in functional outcome. In case of two-part fractures the implant to be used has to be decided based upon the surgeon’s experience in using a particular implant. Better outcomes and lesser complications can be achieved with good intra-operative anatomical reduction of the fracture irrespective of the implant used.
